# The Effects of the COVID-19 Pandemic on Vaccination Hesitancy: A Viewpoint

**DOI:** 10.3390/vaccines11071191

**Published:** 2023-07-03

**Authors:** Mirko Leonardelli, Federica Mele, Maricla Marrone, Cinzia Annatea Germinario, Silvio Tafuri, Lorenza Moscara, Francesco Paolo Bianchi, Pasquale Stefanizzi

**Affiliations:** 1 Section of Legal Medicine, Department of Interdisciplinary Medicine, University of Bari “Aldo Moro”, 70124 Bari, Italy; mirkoleonardelli@gmail.com; 2 Interdisciplinary Department of Medicine, Aldo Moro University of Bari, 70124 Bari, Italy; cinziaannatea.germinario@uniba.it (C.A.G.); silvio.tafuri@uniba.it (S.T.); lorenza.moscara@gmail.com (L.M.); dr.francesco.bianchi@gmail.com (F.P.B.); pasquale.stefanizzi@uniba.it (P.S.)

**Keywords:** vaccination hesitancy, COVID-19, vaccination campaigns

## Abstract

Vaccination hesitancy is considered by the World Health Organization as a danger to global health. In recent years, vaccine hesitancy rates to COVID-19 have been studied worldwide. In our study, we aim to provide an overview of the concept of vaccine hesitancy, with regard to the post-COVID era, and to provide prevention and management strategies. A search of the international literature until March 2023 was conducted in the PubMed database. The 5723 papers found were divided into two groups: prior to the COVID-19 era and from 2021 onward. Papers about the vaccine hesitation phenomenon are becoming more common during the SARS-CoV-2 pandemic and following the marketing that the vaccine companies have carried out on the different types of COVID-19 vaccines. It is advisable that healthcare authorities, at the national and international level, as well as healthcare professionals, at the local level, should promote a series of activities to reduce the vaccine hesitancy rate.

## 1. Introduction

The World Health Organization defines “vaccine hesitancy” as the “delay in accepting or refusing immunization” due to the perception of a low disease risk, reduced accessibility and lack of confidence in safety and efficacy [1].

The refusal of vaccine administration may be due either to moral/religious beliefs or to valid medical contraindications (such as history of prior anaphylaxis) [2]. Most vaccine-hesitant individuals in the USA are of pediatric-age individuals, and they are hesitant for non-medical reasons [3]. Vaccine hesitancy is a major public health problem worldwide [4]. Several studies have examined opinions about the safety, efficacy, relevance, and compatibility with religious beliefs of vaccination, demonstrating significant variations among countries and changes in attitudes toward vaccines over time [5,6]. Vaccines are considered unsafe more in Europe than in the rest of the world: 36% of interviewees in Bosnia-Herzegovina and 41% in France doubt the safety of vaccinations [5]. These data are in line with the large prevalence of vaccine hesitancy worldwide, which increased annually between 2014 and 2016 [7]. A significant number of parents choose to forego vaccinating their children or request that vaccines be administered according to a different vaccination schedule [8,9]. Despite this, the overall prevalence of complete refusal of the vaccine schedule is still low (about 1% of children under the age of 3 in the USA) [10]. 

The scientific classification of vaccines opinions is challenging due to diverse attitudes and beliefs. Surveys have shown between 50 and 60% of respondents in different studies strongly believe that vaccinations are safe and essential [11,12,13,14]. Lack of trust in the government or health professionals leads individuals to turn to “alternative medicine”, refusing other medical treatments [15,16,17]. About 60–70% of vaccination exemption requests are motivated by concerns about safety and adverse effects [18]. Concerns about vaccine safety include side effects like Guillain-Barré syndrome, intussusception, and pain, as well as potential immune-system overload and infections [19,20,21,22]. 

An important role in spreading fears about vaccine safety is played by negative media messages and word of mouth among people [22,23,24]. Some fears about vaccine safety are justifiable, while others have no scientific basis [25,26]. Vaccine hesitancy results in an increased risk of diseases that can be prevented by vaccination in the unprotected population [27]. Unvaccinated individuals have an approximately nine-times greater risk of contracting chickenpox, up to 35 times greater risk of contracting measles, and 6 to 28 times greater risk of contracting whooping cough [28,29,30]. Vaccine-preventable disease outbreaks not only spread preventable diseases in the community, but also consume public health resources [31,32,33]. 

Vaccine hesitancy is a significant barrier to vaccination coverage, affecting herd immunity and community transmission. In recent years, this is also applicable even to the Sars-CoV-2 pandemic [34]. Although there has been a decrease during the different pandemic years, vaccine hesitancy rates in the US remain high [35]. An age of under 60 years, low educational level, low household income, residence in rural areas and lack of health insurance are the factors most correlated with vaccine hesitancy regarding COVID-19 [36,37,38]. Centers for Disease Control stated that lack of confidence in the vaccination process, and concern about vaccine safety and adverse effects, were the main justifications for COVID-19 vaccine-uptake refusal [36]. The main deterrents to the administration of the bivalent booster of COVID-19 vaccine include ignorance of eligibility requirements, unawareness of the availability of the booster dose, the idea that they are still protected, and concerns about the safety and efficacy of the booster [39]. These results demonstrate how the information of the patients about new vaccination proposal is important for healthcare professionals. 

Critics argue that concerns about vaccine safety, lack of health information, ignorance of virus mechanisms of action, doubts about vaccine efficacy against emerging variance, and erroneous myths about serious side effects contribute to the high rate of reluctance in COVID-19 vaccine administration. Understanding the causes of these resistances is crucial for reducing the spread of infection and reducing related pathology.

Due to the the recentness of the issue, to the best of our knowledge there is scarce literature on this topic. Therefore, although this paper is not a systematic review, the aim is to focus the attention of the readers on the effects of vaccine hesitancy on public health, analyzing whether the concept of vaccine hesitancy has become more or less widespread in the post-COVID-19 era.

## 2. Methods

To find relevant scientific publications in English, a search of the international literature was conducted from January 2023 until March 2023 in the PubMed and Scopus electronic databases, using the keywords “Vaccination” and “Hesitancy”. Considering the large number of articles published, we did not add other keywords. To identify papers that assessed the incidence of vaccine hesitancy and potential methods of counteracting it, all articles were filtered by title and abstract content. All the types of papers with those keywords were included within the research (original articles, review, commentaries, editorials, opinions). In addition, searches were conducted to verify whether the bibliographic references of the various articles included in the analysis sample were relevant to the present article. In order to collect as many papers as possible, inclusion criteria included articles published in English and full text or only abstract available. Articles published not in English were excluded. This paper was not a Systematic Review, so we did not strictly follow the PRISMA statement or other guidelines for reviews. Furthermore, since the major aim was to analyze whether vaccine hesitancy has become more or less widespread in the post-COVID-19 era, we focused the attention on the total number of articles in the pre- and post-COVID period and we read only the most relevant articles more carefully.

## 3. Results

The methodological criteria applied resulted in the collection of a total of 5723 scientific articles published in the time interval between 1968 and 2023. Subsequently, the publication year 2021, corresponding to the start of the vaccine campaign for Sars-CoV-2, was defined as the dividing time. In this way, the scientific articles were divided into two groups: one concerning publications prior to the COVID-19 era (totaling 1160 scientific articles) and one concerning publications from 2021 onward (totaling 4563 scientific articles).

## 4. Discussion

Vaccination refusal increases the chance of occurrence of diseases that could be prevented by vaccination in the general population [40]. The World Health Organization considers vaccination hesitancy a danger to global health [41]. There are several approaches to removing the misconceptions about vaccination. For example, during the Polio vaccine campaigns, some individuals claimed that the immunization was prohibited since it contained pig protein, which is not “halal” (permitted to eat). Later, a separate Islamic scholar claimed that it is “halal” for Muslims to receive the polio vaccine [42,43]. Similar to this, various false information concerning Ebola and treatment initiatives was shared during the previous Ebola outbreak [44]. Various initiatives have been made to raise vaccination rates and eliminate vaccine-related myths. For instance, recruiting female community workers, religious leaders, social activists, and social media to promote immunization [45].

In several countries, COVID-19 vaccination was licensed for use in the general population between late 2020 and early 2021. Vaccine hesitancy rates regarding the COVID-19 vaccine by the general population have been studied worldwide and are relatively well defined [46]. For example, in a recent study conducted by Sallam M., some countries in Asia, in particular China, demonstrated the highest rates of acceptance of COVID-19 vaccine administration by the general population, with values even higher than 90% for the latter, compared to other countries in the world that reported lower rates, for example in several European and North American countries, including Italy, where acceptance values below 60% were recorded for the latter [47]. 

Several studies conducted among the general population, have found that the severity of consequences related to Sars-CoV-2 infection, as well as some sociodemographic factors including gender, age, education, income, and occupation, strongly influence COVID-19 vaccination hesitation rates [48]. A thorough understanding of the causes of this resistance is critical. Concerns about the safety of vaccines in the elderly and patients with various pre-existing comorbidities, insufficient health information, ignorance of the mechanisms of action of the virus, doubts about the efficacy of available vaccines against emerging Sars-CoV-2 variants, and erroneous myths about some serious side effects of COVID-19 vaccines are the main reasons behind the high rate of reluctance in COVID-19 vaccine administration [49]. It should be noted that, unexpectedly, the results highlighted by some clinical trials regarding the efficacy of the COVID-19 vaccine have led to a drastic reduction in this reluctance, allowing a significant reduction in the spread of infection and the severity of related pathology [50]. 

In addition, refusal or delay in COVID-19 vaccination has been linked to age, literacy rate, lack of interest in the vaccine, and disbelief in healthcare and in management of the Sars-CoV-2 pandemic [51]. Surprisingly, the literature reported data from the acquisition of surveys submitted to the general population that revealed a high level of distrust of vaccines and of fear about side effects resulting from vaccine administration in the category of medical students [52]. 

Therefore, in our opinion, vaccine hesitancy has become highly prevalent following the marketing of COVID-19 vaccines. This opinion is supported by the results of our literature research: comparing the pre- and post-COVID period, the percentage of publications on the theme of vaccine hesitancy is dramatically increased, as shown in Table 1. It can thus be assumed that the need for maximum dissemination of prevention campaigns and techniques to manage the issue related to vaccine hesitancy developed due to COVID-19. 

Key recommendations include establishing constructive dialogue, identifying patient concerns, providing targeted responses to those concerns, and highlighting all the benefits related to full adherence to the immunization program [53]. A systematic review in 2015 on vaccine hesitancy management techniques reported few initiatives explicitly designed to address this form of reluctance or to measure the impact of management intervention, demonstrating that a multidisciplinary, communication-informed approach appears to be the most effective management method [54]. To achieve the intended goal, it is essential to initiate a constructive, nonconfrontational discourse that already assumes complete adherence to vaccine treatment [55]. Objectives to be pursued in building communication include identifying concerns that afflict the patient and recognizing external factors that influence the patient’s knowledge and attitudes toward the vaccine [56]. Patient acceptance of vaccination programs may depend on how the healthcare provider exposes information related to them. In two observational studies, it was shown how greater adherence to the vaccine proposal was achieved through presumptive communication rather than through a participatory approach [57,58]. 

Identifying the fears of patients who are to undergo vaccination and recognizing the factors that feed these fears is a key objective that must be sought in structuring productive communication [59]. Healthcare providers must be aware that different patients may give more weight to anecdotal information about vaccination risks by extrapolating from alternative sources to scientific studies, such as the mass media [60]. There are many concerns related to vaccine administration that can be discussed and debated. To encourage interest in and confidence in COVID-19 vaccines, for instance, websites, social media, and leaflets/posters can assist healthcare practitioners when discussing immunizations [61].

Some patients may need to receive information from a variety of sources; therefore, the healthcare professional should address in detail the hesitations that arise during the interview, reiterating that vaccination is safe and effective, and that the failure to vaccine administration may result in serious illnesses for family members and the patient themselves [62]. The arguments made by health professionals should be tailored to the type of patient to whom they are addressed. One of the potential issues that should be addressed concerns the risk and benefits related to vaccine administration. Vaccine immunization is globally recognized as one of the best preventive health interventions [63]. However, it is critical to recognize the limitations of vaccination because there is no 100% guarantee of its safety and efficacy. Although this information may undermine the trust of patients in vaccination campaigns, it is important that the information is provided in the correct manner, especially when communication occurs with patients who tend to overestimate the side effects of the vaccine and underestimate, instead, the risks of the disease [64]. 

It is crucial to dispel false myths, to correct information and to direct patients to reliable data sources by avoiding the use of language that is inaccessible to patients or scientific terms that are difficult to understand [65]. Several scientific studies advise healthcare providers to justify the unfoundedness of misinformation about vaccine administration, to convey concise and direct concepts, and to avoid instilling fear by emphasizing the danger of non-vaccination since this approach could result in increased risk perception [66]. 

Techniques to prevent the phenomenon of vaccine hesitancy have not yet been concretely defined since there are no sufficiently significant studies on this issue in the literature. The usefulness of the vaccination campaign should be argued openly, honestly, and non-confrontationally by physicians through information meetings prior to vaccine administration since health professionals are a reliable source of information about vaccines [67]. Health education should be comprehensive: health professionals should offer clear, evidence-based information, avoiding complex statistical data and directly answering questions posed by patients. Creating fact sheets on vaccines may be enough to dispel the doubts of most patients, although some of them may need to receive more in-depth scientific information, or they may find more useful some pamphlets containing direct questions and answers or direct testimonials from other patient who supports the vaccine campaign [68]. 

The limit of this study is the use of only English-language articles. Possible bias may have occurred since this article may not have included the perspectives of people from non-English speaking populations, thereby limiting the understanding of vaccines in them. In addition, further research could be conducted on specific populations, such as minorities, to better determine the strategies that can be used to overcome vaccine hesitancy.

Further studies should analyze some issues that emerged in our study, for instance, the strategies to mitigate vaccine hesitancy through regional, national, and international campaigns. Another future topic should be related to the public health concerns with vaccine hesitancy, such as the possible reintroduction of previously eradicated diseases, delay in their eradication, persistence of pathogen circulation, and impact on healthcare systems and on human personnel.

## 5. Conclusions

In our study, the search of the international literature about vaccine hesitancy has demonstrated that this is an attitude that has become globally prevalent also as a result of the spread of SARS-CoV-2 pandemic and following the marketing of the various types of COVID-19 vaccine. 

From our analysis of the literature, it is advisable that the healthcare authorities, at the national and international levels, as well as the health professionals at the local level should implement a series of prevention, management and promotion activities aimed at reducing the vaccine hesitancy rate in the population.

## Figures and Tables

**Table 1:** Scientific articles about vaccine hesitancy.

Period	Number of Articles	Percentage
Pre-COVID-19 ^1^	1160	20.27%
Post-COVID-19 ^2^	4563	79.73%

^1^ From 1968 to 2020. ^2^ From 2021 to 2023.

## Data Availability

Not applicable.
